# Development of mouthparts in the cicada *Meimuna mongolica* (Distant): successive morphological patterning and sensilla differentiation from nymph to adult

**DOI:** 10.1038/srep38151

**Published:** 2016-11-30

**Authors:** Yanan Hao, Christopher H. Dietrich, Wu Dai

**Affiliations:** 1 State Key Laboratory of Crop Stress Biology for Arid Areas, College of Plant Protection, Northwest A&F University, Yangling, Shaanxi 712100, China; 2 Key Laboratory of Plant Protection Resources and Pest Integrated Management of the Ministry of Education, College of Plant Protection, Northwest A&F University, Yangling, Shaanxi 712100, China; 3 Illinois Natural History Survey, Prairie Research Institute, University of Illinois at Urbana-Champaign, 1816 S. Oak St., Champaign, IL 61820, USA.

## Abstract

Development of the mouthparts in the cicada *Meimuna mongolica* (Distant) is investigated here for the first time using scanning electron microscopy in order to document changes occurring in different nymphal instars and from nymph to adult, during which a shift from subterranean root-feeding to feeding on aboveground parts of the host plant occurs. The structure and component of mouthparts is similar to those found in other hemipteran insects. Fourteen types of sensilla and five types of cuticular processes were found on the mouthparts of nymphs and adults. Significant general transformations during development include changes in: (a) the size and shape of the labrum from square to long and shovel-shaped; (b) increases in type and quantity of sensilla with the stage of development; (c) the ridges at the tips of the mandiblar stylets become more prominent in later stages of nymphal development, while odontoid protrusions more prominent in the female than in the male of the adult; and (d) the cross section of the stylets is subcircular in nymphal stages but oblong elliptical in the adult. The implications of these mouthpart transformations on the feeding ability of nymphs and adults and their possible relationship to the feeding niche are discussed.

Evolutionary adaptations for the nutritional exploitation of host plants represent a major force driving the diversification of phytophagous insects. Mouthparts of insects differ according to differences in feeding behavior and habits and bear important sensory and feeding structures that are crucial for host plant recognition and for obtaining food from host plant tissues. Hemiptera, a very large and diverse insect order, are united by their specialized piercing-sucking mouthparts by which they feed on the fluid contents of various host tissues.

Several previous studies have examined the mouthpart morphology of adult Hemiptera based on light and scanning electron microscopy^1^,^2^, including as Aphidoidea^3^,^4^,^5^, Psyllidae^6^,^7^, Aleyrodidae^8^,^9^, Cicadellidae^10^,^11^,^12^,^13^ and Fulgoroidea^14^,^15^. These studies demonstrated that the structures of the mouthparts vary between different species and that some such differences are related to differences in feeding behavior.

Relatively few previous studies focused on the mouthpart sensilla of immature insects, with most focusing on Holometabola, in which adults and immatures usually occupy entirely different feeding niches. Studies of Coleoptera^16^,^17^, Diptera^18^, Dermaptera^19^, Lepidoptera^20^,^21^ and Mecoptera^22^, have highlighted dramatic differences in larval mouthpart morphology related to different feeding habits^22^. Unfortunately, mouthpart morphology of hemipteran nymphs remains little studied. Because nymphs and adults of Hemiptera usually share the same feeding niche, it is generally assumed that the mouthparts of nymphs resemble those of adults. However, in cases where a shift in feeding niche occurs between the nymphal and adult stage, morphological differences between the mouthparts of nymphs and adults may be expected.

Cicadas (Hemiptera, Cicadidae) are a diverse family of sap-sucking hemipterans well known for their loud and species-specific courtship songs. Some are considered horticultural pests due to the twig dieback that may be caused when large numbers of females insert eggs into trees and shrubs. Further injury caused by their feeding usually goes undetected since their nymphs are long-lived and occur underground^23^. Nymphs feed on xylem of plant roots underground^24^, while adults feed exclusively on xylem fluid from the branches of their host plants^25^,^26^,^27^. Nymphs and adults differ substantially in external morphology in part due to their different ecological niches^27^ but the differences in mouthparts have not been studied in detail previously.

The cicada *Meimuna mongolica* (Distant) is widely distributed in the southern Palaearctic and northern Oriental Regions (e.g., North Korea, South Korea, Mongolia, and China). It is one of the most important pests of economic forest in the Guanzhong Plain of Shaanxi Province, China, which lies north of the Qinling Mountains. Previous studies on *Meimuna mongolica* were mainly focused on adult morphology and taxonomy^28^, and the morphology or morphometrics of the final instar exuviae^29^. Nymphs remain little studied^27^,^30^ because they are subterranean and difficult to obtain.

Research on mouthpart morphology is needed to provide insights into feeding mechanisms^31^ and to identify traits useful for classification and identification^32^. This paper describes developmental trends in the mouthparts of *Meimuna mongolica*, with emphasis on the structure and topographic position of the mouthpart setae from the first instar nymph to adult stages. Implications of the mouthpart transformations on the feeding abilities of the cicada during development are discussed.

## Results

### Gross morphology of mouthparts

**Figure 1:**
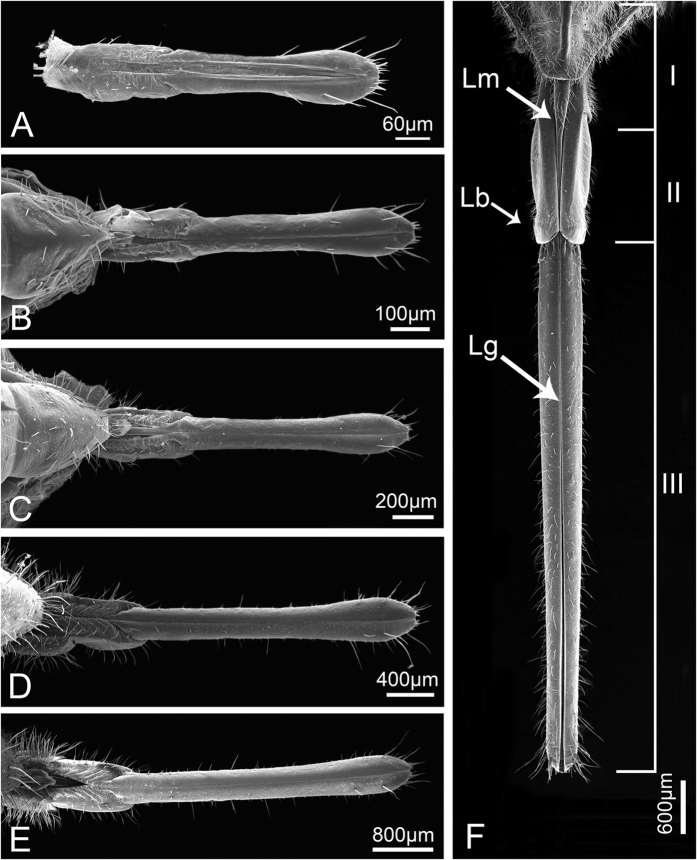
Scanning electron micrographs of *Meimuna mongolica* mouthparts in dorsal (anterior) view showing their overall morphology. (**A–E**) First to fifth instar nymph, respectively. (**F**) Adult. Lm, labrum; Lb, labium; Lg, labial groove; I-III, the first to third segment of labium.

**Figure 2:**
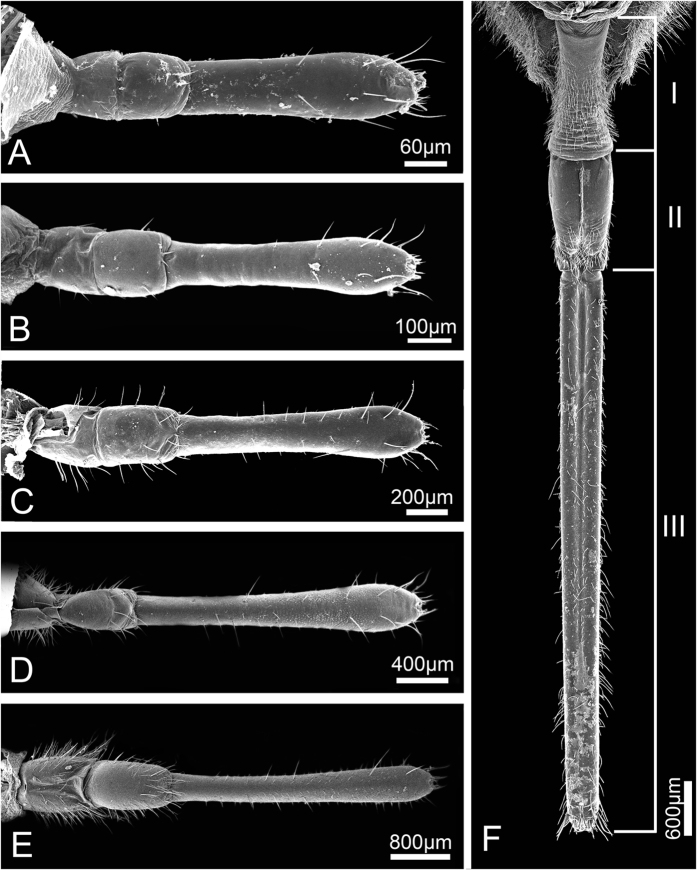
Scanning electron micrographs of *Meimuna mongolica* mouthparts in ventral view showing their overall morphology. (**A–E**) First to fifth instar nymph, respectively. (**F**) Adult. I-III, the first to third segment of labium.

The head of *Meimuna mongolica* (Distant) is shaped like an inverse triangle and is densely clothed with setae. As in other Auchenorrhyncha, the mouthparts, on which various kinds of sensilla are distributed, arise from the posterior part of the head capsule and consist of a relatively small labrum (Lm) and a tubular labium (Lb) subdivided into three different length segments (Figs 1 and 2). An infolding on the dorsal surface (appearing ventral due to the opistognathous orientation of the mouthparts) of the labium forms a deep longitudinal labial groove (Lg) (Fig. 1F), within which lies the stylet fascicle (Sf), consisting of two inner maxillary stylets (Mx) completely surrounded by two mandibular stylets (Md). The basic mouthpart components of nymphs and adults are the same, while their lengths and some specific structural details are quite different.

### Morphological characters of the sensilla and cuticular processes on the mouthparts

Based on their morphology six kinds of sensilla and three kinds of cuticular processes were identified on the mouthparts of *Meimuna mongolica*, and some of them can be subdivided into groups on the basis of size and distribution, so in general, fourteen types of sensilla and five types of cuticular processes can be identified.

**Figure 3:**
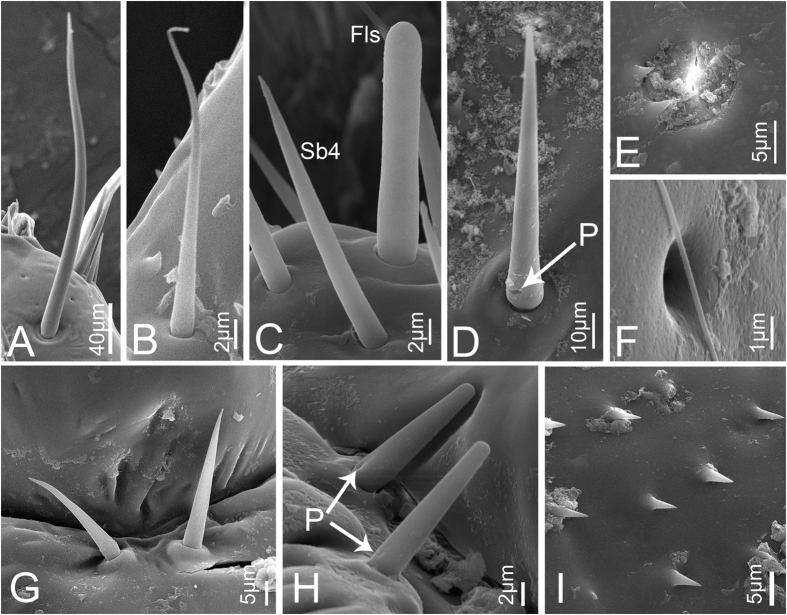
Sensilla and cuticular processes on mouthparts of *Meimuna mongolica* nymphs. (**A**) Sensilla trichodea I in fifth instar. (**B**) Sensilla trichodea II in first instar. (**C**) Finger-like sensilla (Fls) and Sensilla basiconica IV (Sb4) in first instar. (**D**) Sensilla basiconica I in fifth instar. (**E**) Sensilla coeloconica I in fifth instar. (**F**) Poriform sensilla in fourth instar. (**G**) Sensilla basiconica III in second instar. (**H**) Sensilla basiconica II in second instar. (**I**) Microtrichia in fifth instar. P, pore.

**Figure 4:**
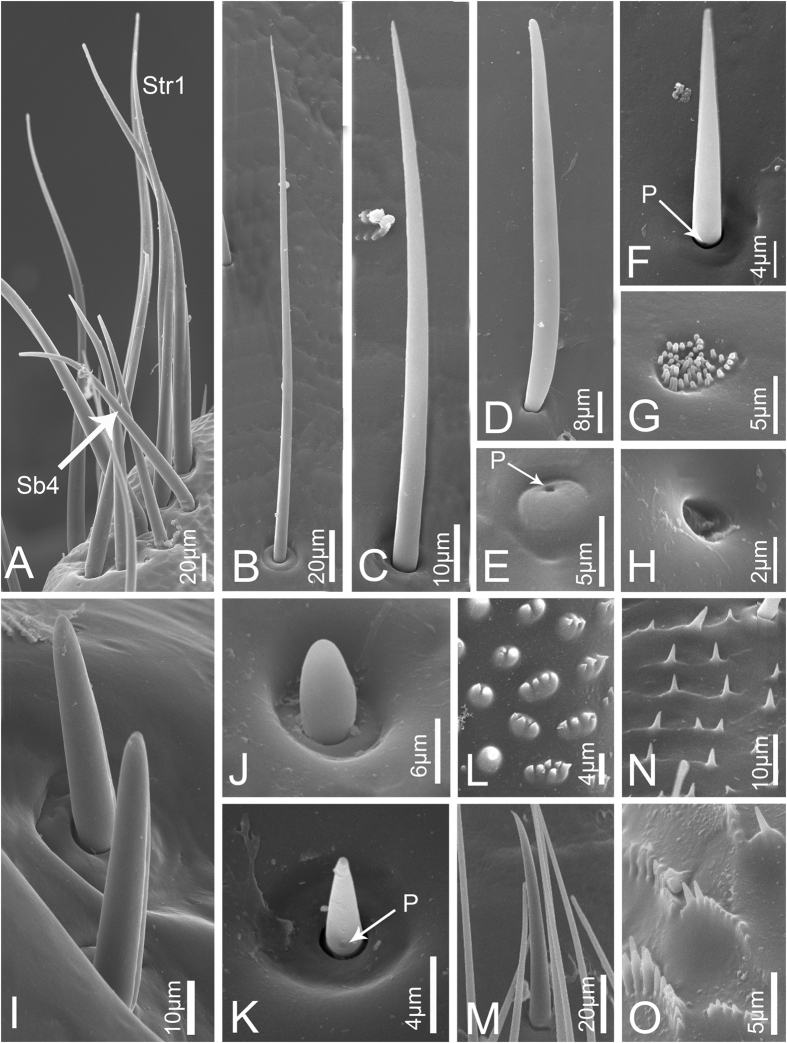
Sensilla and cuticular processes on mouthparts of *Meimuna mongolica* adults. (**A**) Sensilla trichodea I (Str1) and Sensilla basiconica IV (Sb4). (**B**) Sensilla trichodea II. (**C**) Sensilla trichodea III. (**D**) Sensilla trichodea IV. (**E**) Hemispheric sensilla. (**F**) Sensilla basiconica I. (**G**) Sensilla coeloconica I. (**H**) Poriform sensilla. (**I**) Sensilla basiconica II. (**J**) Sensilla coeloconica II. (**K**) Sensilla basiconica IV. (**L**) Mammillary processes. (**M**) Sensilla basiconica III. (**N**) Microtrichia. (**O**) Scaly structure. P, pore.

**Sensilla trichodea I (Str1)** are relatively long and stout, inserted in a round sunken socket, with smooth cuticle, thick at the base, tapered and slightly curved in the apical half (Fig. 3A). Only a few can be found at the end of the third labial segment in nymphs. In adults, each sensillum has a longitudinal groove extended from base to apex and the sensilla are more numerous and grouped into a cluster (Fig. 4A). Their length and basal diameter gradually increase from the first to the fifth instar, while in adult, their sizes are similar to those found in the fourth instar (Table 1).

**Figure 5:**
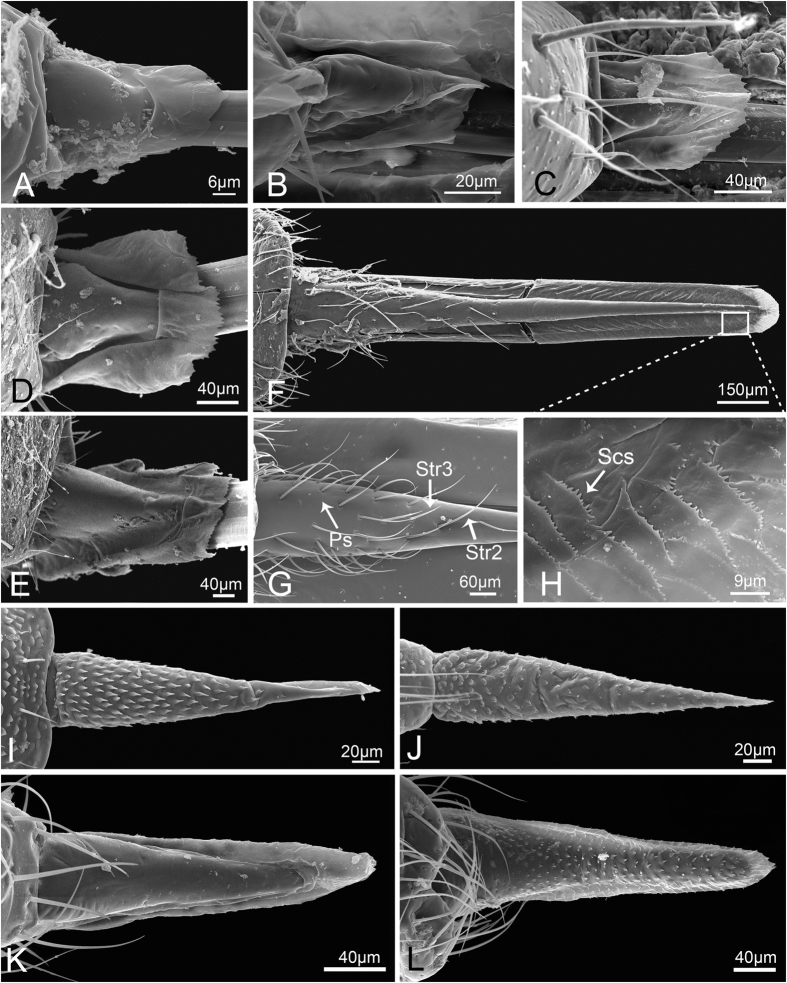
Labrum morphology of *Meimuna mongolica* and other insects in Cicadomorpha. (**A–E**) First to fifth instar nymph, respectively. (**F**) Adult of *Meimuna mongolica*. (**G**) Enlarged view of the base of labrum showing various sensilla. (H) Partially enlarged view of white box in (**F**) showing Scaly structure on margin of labrum. (**I**) The labrum of *Atkinsoniella opponens* (Walker) (Cicadellidae). (**J**) The labrum of *Gargara* sp. (Membracidae). (**K**) The labrum of *Eoscarta seimblndi* Lallemand (Cercopidae). (**L**) The labrum of *Aphrophora bipunctata* Melichar (Aphrophoridae). Str2, sensilla trichodea II; Str3, sensilla trichodea III; Ps, poriform sensilla; Scs, scaly structure.

**Sensilla trichodea II (Str2)** are shorter and thinner than Str1, inserted in a pit with a round convex socket, slender, with smooth cuticle (Figs 3B and 4B), and widely distributed on each labial segment. In adults, Str2 are also present on the labrum (Fig. 5G). Their length and basal diameter gradually increase with increasing instar in nymphs but slightly decrease between fifth instar and adult (Table 1).

**Sensilla trichodea III (Str3),** which only appear in adults, are inserted in a pit with an inconspicuous sunken socket, are quite straight, with smooth cuticle and a sharp tip (Fig. 4C), and are widely distributed on the labrum (Fig. 5G) and each segment of the labium.

**Sensilla trichodea IV (Str4)** also only appear in adults and are inserted with no socket, have a thin base, are slightly thicker in the middle and are blunt at the apex (Fig. 4D). They are mainly distributed on the lateral surface of the first two segments and the first half of the third labial segment.

**Figure 6:**
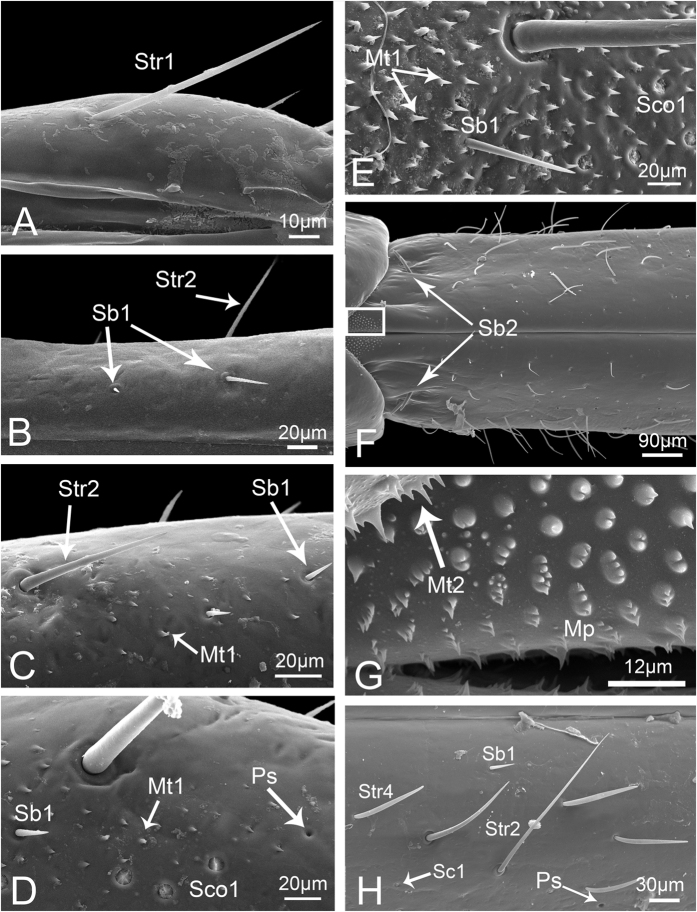
Sensilla and cuticular processes on the labium of *Meimuna mongolica*. (**A–E**) Partial view of third segment of labium, first to fifth instar nymph, respectively. (**F**) Base of third labial segment of adult. (**G**) Enlarged view of white box of (**F**) showing Mt2 and Mp. (**H**) Enlarged view of middle of third labial segment. Sb1, sensilla basiconica I; Sb2, sensilla basiconica II; Str1, sensilla trichodea I; Str2, sensilla trichodea II; Str4, sensilla trichodea IV; Mt1, microtrichia I; Mt2, microtrichia II; Ps, poriform sensilla; Sco1, sensilla coeloconica I; Mp, mammillary processes.

**Sensilla basiconica I (Sb1)** are inserted in a pit with a round concave socket, have smooth cuticle, and are thick at base and sharp at the tip, with a pore at the base (Figs 3D and 4F). They are mainly distributed on the dorsal surface of the labium (Fig. 6B–E,H). Their mean lengths and basal diameters gradually increase from the second to the fifth instar nymphs but slight decrease in adults (Table 1).

**Sensilla basiconica II (Sb2)** are inserted in a pit with a slightly convex socket, have smooth cuticle, and have a pore at the base in nymphs (Fig. 3H) and a longitudinal groove on one side from base to apex in adults (Fig. 4I). They have a thick base and a blunt tip and are mainly distributed in pairs on the dorsal surface of the joint of the second and the third labial segment, usually oriented toward the second segment. Only two pairs occur on both sides of the labial groove (Fig. 6F). Their lengths gradually increase in nymphs, while adults were similar to the fourth instar (Table 1).

**Sensilla basiconica III (Sb3)** have a cylindrical base inserted in convex socket, have a nonporous, smooth cuticular wall, and a sharp point at the tip. Only one pair is present on the ventral surface of the second labial segment, oriented toward the third segment (Figs 3G and 4M).

**Sensilla basiconica IV (Sb4)** are similar to Sb1, while they have no pore at base and sometimes curve at the tip (Figs 3C and 4A). Their cuticles are smooth and distributed only at the sensory field of labial tip. Their lengths gradually increase from the second nymphal instar to adult (Table 1).

**Sensilla basiconica V (Sb5)** only appear in adults. They are quite short and straight, inserted in an obvious slightly concave pit, mostly oriented toward the tip of labium, and have a smooth surface and a pore at base (Fig. 4K). They are distributed on the dorsal surface of the first two labial segments.

**Figure 7:**
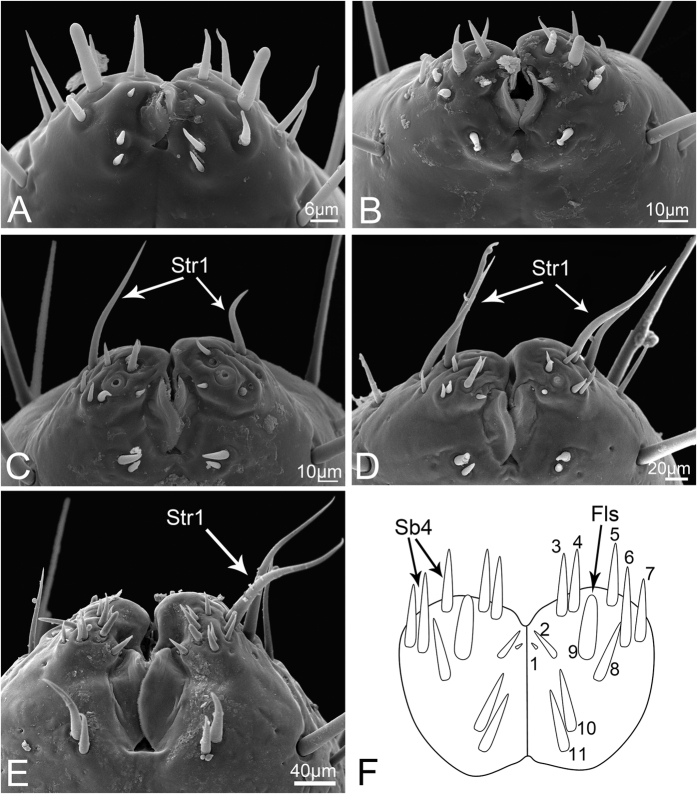
Apex of third labial segment of nymphs *Meimuna mongolica*. (**A–E**) Labial apex of first to fifth instar, respectively. (**F**) Diagram of distal end of labium to show the constant sensilla in nymph. Numbers represent individual sensilla identified. Str1, sensilla trichodea I; Sb4, sensilla basiconica IV; Fls, finger-like sensilla.

**Figure 8:**
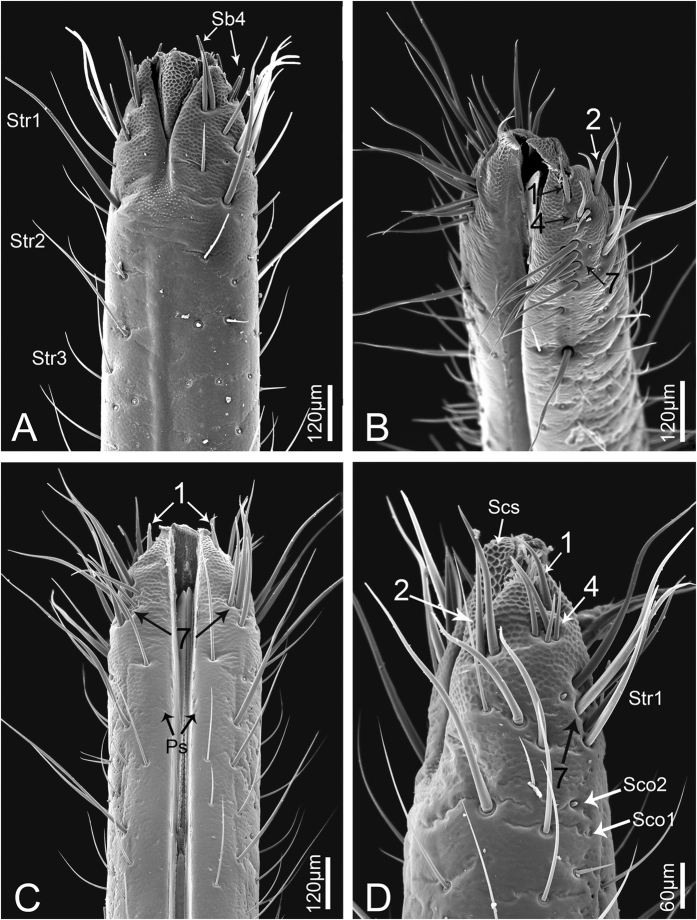
Apex of third labial segment of adult *Meimuna mongolica*. (**A**) Ventral surface. (**B**) View from above. (**C**) Dorsal surface. (**D**) Lateral surface. Numbers of sensilla distributed in each cluster are indicated. Str1-3, sensilla trichodea I-III; Sb4. sensilla basiconica IV; Ps, poriform sensilla; Sco1, sensilla coeloconica I; Sco2, sensilla coeloconica II; Scs, scaly structure.

**Finger-like sensilla (Fls)** are only present in nymphs and are peglike, inserted in a convex socket, and have a smooth surface (Fig. 3C). Only one pair is present in the dorsal sensory field at the apex of the third labial segment in nymphs. The morphology and position are constant from the first to the fifth instar (Fig. 7A–F), but they disappear during the final molt to the adult stage (Fig. 8A–D).

**Sensilla coeloconica I (Sco1)** are a cluster of fingerlike structures arranged in a round concavity. The number of fingerlike structures varies in different sensilla and they are widely distributed on the dorsal surface of the three labial segments in adults (Fig. 4G). In nymphs, Sco1 appear after the third instar but the fingerlike structures are absent or hidden by secretions (Fig. 3E).

**Sensilla coeloconica II (Sco2)** are only present in adults. They are cylindrical, inserted in a round sunken socket, have smooth cuticle, and are mainly distributed on the third labial segment (Fig. 4J).

**Poriform sensilla (Ps)** are visible on the surface as a deeply concave pore (Figs 3F and 4H), first appear after the third instar, and are widely distributed on the labrum and the dorsal surface of the labium in adults, but in nymphs, they can only be found on the labium.

**Hemispheric sensilla (Hs)** are hemispheric, within a slightly sunken socket, have smooth cuticle, and generally have a pore on one side (Fig. 4E). They are only distributed on the distal part of the third labial segment of adults.

**Microtrichia (Mt) are** small rigid projections occurring singly or in groups of two or three arranged together (Figs 3I and 4N), can be divided into three types according to their position. Mt1 occur after the third instar, are widely distributed on the labium of nymphs (Fig. 6C–E) and the lateral side of the first labial segment of adults. Mt2 are mainly distributed on the dorsal side of the junction of the second and third labial segment of adults (Fig. 6G). Mt3 mainly occur on the ventral side of the second labial segment of adults.

**Scaly structures (Scs)** only appear in adults and consist of clusters of spines arranged in scale-like rows (Fig. 4O). They are mainly distributed on labrum and the distal end of the third labial segment (Fig. 5H).

**Mammillary processes (Mp)** only appear in adults and consist of an intumescent base bearing one or more blunt or sharp-tipped projections (Fig. 4L). They are mainly distributed on the third labial segment (Fig. 6G).

### Developmental trends in mouthparts

The most conspicuous general trends in development are increases in the overall size of the appendages, the number and size of sensilla and cuticular processes, and variety of setal types.

#### Labrum

The labrum is a flat structure with conical apophysis in the middle throughout the length. Its size increases with age and the structure differs significantly between nymphs and adults (Table 2). The labrums of nymphs are nearly square and lack obvious sensilla, and the serrations on the terminal margin become more and more numerous from the first to the fifth instar (Fig. 5A–E). The labrum of adults is greatly elongated compared to that of nymphs (Fig. 5F) and has various kinds of sensilla and cuticular processes situated on the apophysis, including Str2, Str3 and Ps (Fig. 5G), which are all absent in nymphs. A flat lateral portion that normally lies concealed inside the labial groove has the cuticle wrinkled and covered with scalelike structures (Scs) at the distal end (Fig. 5H). This overall structure is similar to that found in other Cicadoidea but differs from the more conical labrums of other Cicadomorpha (Fig. 5I–L).

#### Labium

**Figure 9:**
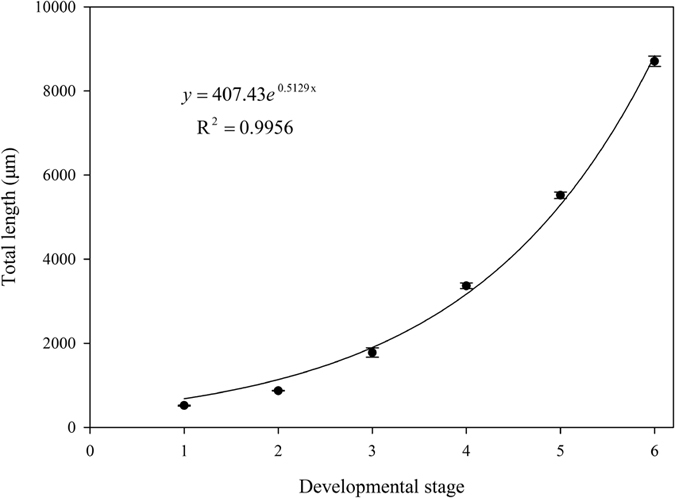
Regression relationship between total length of mouthpart and developmental stages.

**Figure 10:**
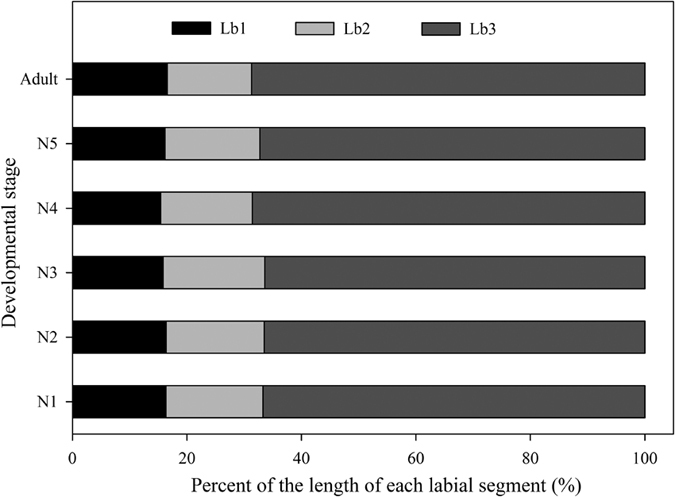
Percent of the length of each labial segment in different instar. Lb1, 2, 3, the first, second, third labial segment.

The modified labium is a tube-like structure, subdivided into three segments (Fig. 2). The total length of the mouthparts differ significantly between developmental stages (*F*_(5,22)_ = 1447.69, *P* = 0.00), and increase exponentially from the first instar nymph to adult (Fig. 9). In all developmental stages, the length and width of each segment differ significantly among instars (Table 2), and the proportion of each segment to the total length is almost the same (Fig. 10).

The shape of labium gradually changes with the developmental stage. In young nymphs, the labium is stout but in late-instar nymphs it becomes thinner and longer. In all nymphal instars, the first two segments are nearly elliptic, and the third segment is oblong-elliptic with an expansion in the subapical region (Fig. 2A–E). In adults, the first segment is wide at both ends and thinner in the middle. The second segment becomes thinner from the base to the tip, and the third segment is almost the same shape but more slender without an expansion at the tip. Furthermore, an indentation can be seen on the ventral side of the third labial segment in the adult (Fig. 2F).

**Figure 11:**
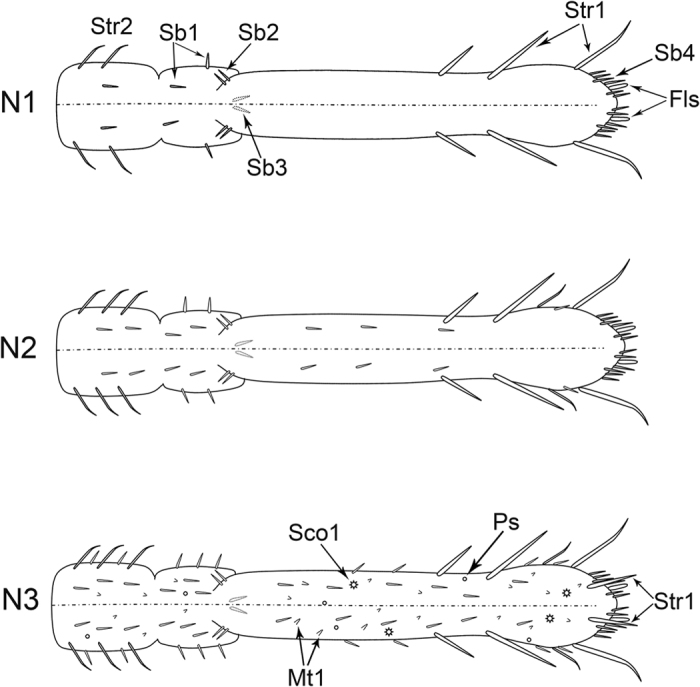
Diagrams of the labium of first three instars showing the distribution and amount of various sensilla. N1, the first instar nymph; N2, the second instar nymph; N3, the third instar nymph. Str1, sensilla trichodea I; Str2, sensilla trichodea II; Sb1, sensilla basiconica I; Sb2, sensilla basiconica II; Sb3, sensilla basiconica III; Sb4, sensilla basiconica IV; Fls, finger-like sensilla; Sco1, sensilla coeloconica I; Ps, poriform sensilla; Mt1, microtrichia I.

**Figure 12:**
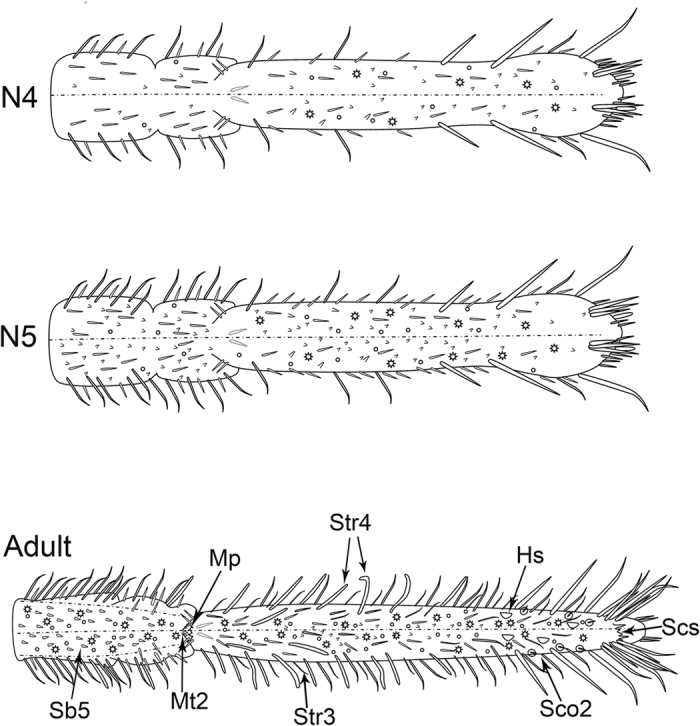
Diagrams of the labium of last two instars and adult showing the distribution and amount of various sensilla. N4, the fourth instar nymph; N5, the fifth instar nymph; Str3, sensilla trichodea III; Str4, sensilla trichodea IV; Sb5, sensilla basiconica V; Mp, mammillary processes; Mt2, microtrichia II; Hs, hemispheric sensilla; Sco2, sensilla coeloconica II; Scs, scaly structure.

The kinds of sensilla and cuticular processes gradually increase from young to older nymphs but the increase is more abrupt between the last nymphal instar and the adult stage (Figs 1, 6A–F, 11 and 12). Str1, Str2, Sb1, Sb2, Sb3, Sb4 and Fls can be found beginning at the first instar, and their distributions are constant during all nymphal stages except for Str2 (Fig. 11). Sco1, Ps and Mt1 appear beginning at the third instar while, at the same time, Str1 appear in pairs on the dorsal surface of the labial tip (Fig. 11, Table 1). In adults, more sensilla and cuticular processes arise, such as Sb5, Str3, Str4, Sco2, Hs, Mp, Mt2 and Scs (Fig. 11, Table 1). Interestingly, Fls disappear in the adult. Sco1, Sco2 and Hs are always distributed on the third segment, and Ps are always present along both sides of the labial groove (Fig. 12). The cuticle of the labial tip is covered by Scs that form a reticulate network (Fig. 8D).

In addition to the type, the numbers of various sensilla change substantially among developmental stages. The two pairs of Sb1 on the dorsal side, one pair of Sb2 on the ventral side, and six pairs of Str1 on the third labial segment (two dorsal, one lateral and three ventral) are constant from nymph to adult (Figs 11 and 12). Notably, a pair of Str1 newly emerges on the dorsal side of the distal end beginning at the third instar (Fig. 7C), and increases to two pairs in the fourth instar (Fig. 7D), three in the fifth instar and seven in adults (Fig. 7E). In addition, more Sb4 can be seen on the ventral side of the adult labial tip. The number of Fls on the labial tip stays the same in all nymphal instars (Fig. 7). The total number of sensilla and cuticular processes gradually increases from the first instar nymph to the adult (Figs 11 and 12).

The morphology of the sensory field at the distal end of the third labial segment is different among developmental stages. From the first to fourth instar nymphs, ten pairs of Sb4 and one pair of Fls can be found in the sensory field and their positions are consistent (Fig. 7A–D), while in the fifth instar, one more pair of Sb4 can be found (Fig. 7E). In adults, so many sensilla are present on the tip of the labium that it is difficult to determine which belong to the sensory field but, in general, four sensilla groups can be distinguished. Two pairs of strong Str1 on the ventral surface are similar to those found in nymphs, however the other three groups are recombined (Fig. 8).

#### Stylet fascicle

**Figure 13:**
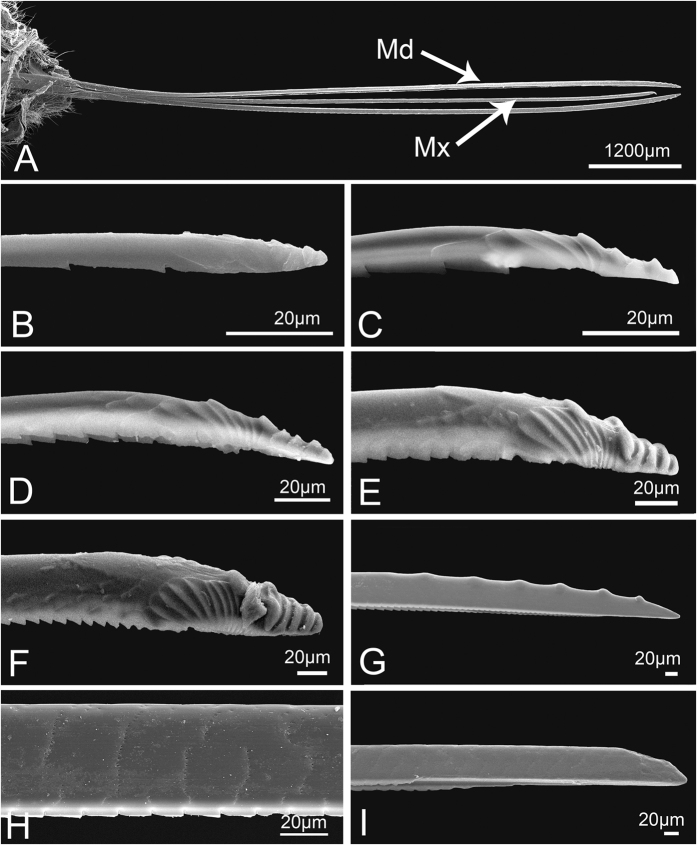
Stylet fascicle and mandibular stylet of *Meimuna mongolica*. (**A**) Whole stylet fascicle of adult showing outer mandibular stylets (Md) and inner maxillary stylets (Mx). (**B–F**) Tip of mandibular stylet of first to fifth instar of nymph, respectively. (**G**) Mandibular stylet of adult female. (**H**) Outer surface of middle part of mandibular stylet showing rows of small pits. (**I**) Mandibular stylet of male adults.

The stylet fascicle (Sf) is an elongate structure composed of two mandibular stylets (Md) and two maxillary stylets (Mx) basically of equal length (Fig. 13A). They originate in the head capsule and are always entirely enclosed within the labial groove when not in use. The Md are located laterally to the Mx and their outer surface is smooth in nymphs (Fig. 13B–F), but has a series of irregular transverse rows of small pits from the base to the proximal part in adult (Fig. 13H). Morphological variation of Md mainly occurs near the apex. From the first to the fifth instar, terminal transverse ridges of Md become more and more evident (Fig. 13B–F). In adults, females differ from males. The apex of the female Md is pointed with several protrusions located at the extreme tip (Fig. 13G), while the Md of the male is blunt and lacks protrusions (Fig. 13I). The edges of the Md in both sexes are odontoid from base to apex.

**Figure 14:**
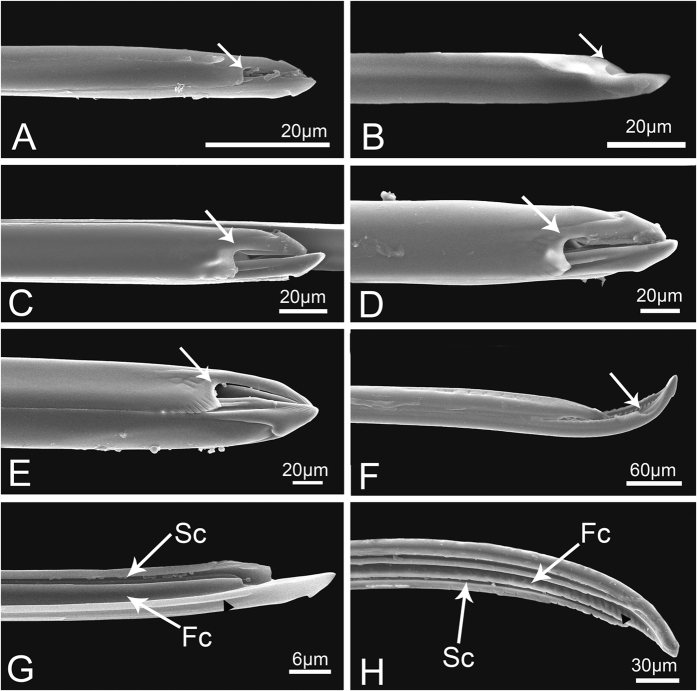
Maxillary stylet of *Meimuna mongolica*. (**A–F**) Apex of interlocked maxillary stylets of first instar nymph to adult, respectively, showing smooth outer surface and breach (white arrows) with adjacent ridges. (**G**) Inner surface of maxillary stylet of the first instar showing food canal (Fc), salivary canal (Sc) and smooth middle ridge (black triangle). (**H**) Inner surface of maxillary stylet of adult showing food canal (Fc), salivary canal (Sc) and middle ridge (black triangle).

The Mx are interlocked, which prevents them from separating during feeding, The outer surface of the Mx is quite smooth except for the distal end, which exhibits some differences between nymphs and adults. The serrated ridges beside the breach deepen gradually from first to fifth instar (Fig. 14A–E), while in adults the ridges smoothly stretch from apex to base (Fig. 14F). The food and salivary canals are formed by the ridges in both nymphs and adults (Fig. 14G,H). These internal ridges of the Mx are quite smooth and few indentations can be seen in first instar nymph (Fig. 14G), but indentations on the middle ridge become more and more evident from second instar nymph to adult (Fig. 14H).

**Figure 15:**
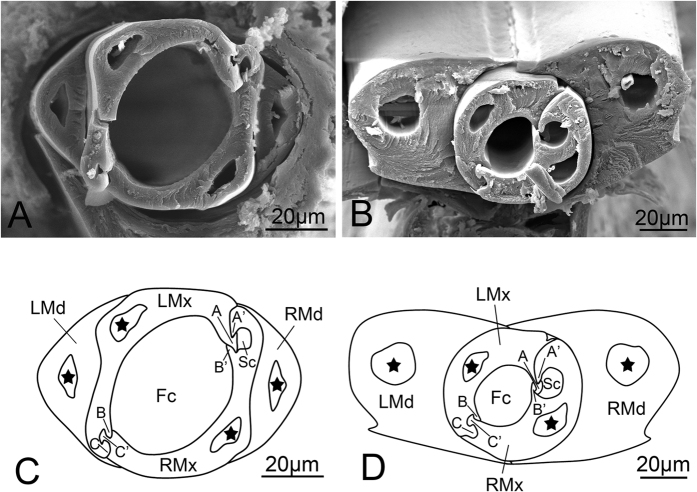
Cross section of stylet fascicle of *Meimuna mongolica*. (**A,C**) Cross section of stylet fascicle of fifth instar nymph. (**B,D**) Cross section of stylet fascicle of adult showing shape of stylets, food canal (Fc), salivary canal (Sc), dendritic canals (black star) and the interlocking mechanism. LMd, left mandibular stylet; RMd, right mandibular stylet; LMx, left maxillary stylet; RMx, right maxillary stylet; A, left T-shaped process; B and C, left hooked process; A’ and B’, right hooked process; C’, right T-shaped process.

The morphology of the stylet fascicle in cross section is similar in different nymphal instars but quite different in the adult. The mandibular stylets are all convex externally and slightly concave internally to form a groove enclosing the Mx, but they are crescent-shaped in nymphs (Fig. 15A,C) and semicircular (Fig. 15B,D) in adults. Each Md has a large circular dendritic canal (Fig. 15) that extends the entire length. The dendritic canals of the nymphal Md are crescent-shaped, while in the adult they are rounded (Fig. 15). The interlocked maxillary stylets are square in nymphs and round in adults. Each Mx has a dendritic canal in the center. The interlocking mechanism consists of hooked processes and T-shaped processes in both nymph and adult, thus forming a large food canal and a smaller salivary canal (Fig. 15). The food canal is much larger than the salivary canal in nymphs but the size difference is less pronounced in adults (Fig. 15).

## Discussion

Highly modified piercing-sucking mouthparts, which play important roles in finding hosts, feeding and, in some cases, transmitting pathogens, are characteristic of Hemiptera. Here we present the first detailed observations of mouthpart development and morphological variation from nymphs to adults in Cicadidae using scanning electron microscopy. The mouthpart morphology of *Meimuna mongolica* is generally similar to that of other hemipterans in basic structure and components^1^,^2^,^9^,^10^,^33^,^34^,^35^,^36^ but detailed comparison to other Hemiptera reveals considerable variation. Our research has revealed that the labrums of Cicadoidea are nearly rectangular flat structures, while in other Cicadomorpha the labrum is conical and triangular or pyramidal^10^,^12^,^37^. Further study of variation in the morphology of the labrum of nymphs may therefore prove useful for classification and identification^38^, as well as for ecological or physiological study.

In Hemiptera, the number of labial segments varies from 1–5 with the usual number being 3 or 4, and only *Lycorma delicatula* (Fulgoridae) and aphids (Aphidoidea) have been reported to have five labial segments^39^. As in most other hemipterans, *Meimuna mongolica* has three labial segments, but the relative lengths the segments differ from those of other groups. In Fulgoromorpha, which feed preferentially on phloem, the first segment is comparatively short and the second two are much longer and subequal in length^39^. In *Meimuna mongolica*, the first two segments are both short but the third is much longer. This structure may provide stronger support, facilitating insertion of the stylets into the xylem of dense woody host plants.

The total length of mouthparts increases exponentially from the first instar nymph to adult *Meimuna mongolica*. This trend is in accordance with previous measurements of the width of the head of *Meimuna mongolica*^27^,^30^. Head capsule width has also been regarded as an important index to distinguish stages of lepidopterous larvae^40^ and other Cicadomorpha^41^,^42^. In North American periodical cicadas, an alternative measurement index has been used to distinguish *Magicicada* nymphal instars, including body length, anterior femora, anterior tibiae and hind tibiae^42^. Because mouthpart length exhibits the same variation trend this measurement may also be used for identification of instars.

Based on the morphological classification systems of Altner and Prillinger^43^ and Brożek^44^, sensilla found on the labium of *Meimuna mongolica* may be classified as fourteen different types. The numbers and types of sensilla increase with increasing nymphal instar, but especially in the transition to the adult stage, and the sizes of the sensilla also change. Only seven kinds of sensilla were found in the first two instars, while Sco1 and Ps begin to appear beginning with the third instar. Up to thirteen kinds of sensilla were found in adults. Constant numbers of sensilla among instars have been found in some other insects, such as lepidopteran larvae^45^. Larger numbers of sensilla have been correlated with broader host ranges^46^. In cicadas, the differences in sensilla between nymphs and adults may be at least partially due to the very different microhabitats inhabited by the different life stages. As in other cicadas, the nymphs of *Meimuna mongolica* live underground. We found them concentrated within the 21–30 cm-deep soil layer^47^, which may harbor the largest numbers of small roots and therefore provide the most abundant food source for cicada nymphs^48^. The more homogeneous subterranean microhabitat of nymphs may partially explain the limited types and numbers of sensilla present on nymphal mouthparts^49^. For adults, a greater variety of sensilla may be necessary to facilitate selection of feeding sites, selecting conspecific mates and finding suitable oviposition sites^50^.

As the first sensory organs to contact plants, sensilla on the tip of the labium play essential roles in host plant identification, and differences in numbers, distribution and types of these sensilla may reflect variation in their feeding behavior and diet breadth. In nymphs, Sb4 and Fls are found in a sensory field and their numbers and position are consistent from the first to the fifth instar. However in adults, their morphology and numbers have changed, including loss of the pair of Fls. It is not clear whether this transition is a result of degeneration or transformation into a different sensilla type. Sb4 in nymphs and Str1 in adults may both function in mechanoreception. Sensilla trichoidea on the labial tip of adults have been reported in Nepomorpha and the number of clusters varies among families^51^. The hemispheric sensilla and poriform sensilla have only one pore and are most likely gustatory or contact chemoreceptive sensilla. Other nonporous sensilla are presumably mechanoreceptive structures^43^,^44^. As indicated by previous studies, assignment of sensilla to functional groups is possible based on the outer cuticular structures such as the presence of a molting pore, but differences in shape are not always in accord with differences in functionally relevant internal structures^43^. Thus, confirmation of sensilla function requires more investigation incorporating study of ultrastructure by transmission electron microscopy.

Previous studies have shown that the mandibular stylets are shorter compared to the maxillary stylets in leafhoppers^35^,^52^, but are nearly as long as the maxillae (93–99%) in planthoppers^52^. In *Meimuna mongolica*, the mandibular stylets appear slightly longer than the maxillary stylets exteriorly in some individuals but the opposite in others. Because the stylets slide longitudinally against each other and assume different relative positions during probing of plant tissues and feeding, such differences are probably due to the differences in relative positions of individuals at the time of capture and fixation. In general, the relation between the length of mandibular and maxillary stylets in cicadas is similar to that of planthoppers.

The sharp end and the protrusions on the apical surface of the mandibular stylets stabilize the movement of maxillary stylets during probing, providing a fulcrum for the movement of the maxillae^1^ and helping anchor the insect to the substrate during ecdysis^11^. The number and size of protrusions on the stylets varies among species of hemipterans^12^,^15^, and may reflect variation in properties (e.g., density) of the host plant tissue^6^. We found that the protrusions on the apical surface of Md become much deeper from the first to fifth instar, which may provide increasingly strong anchoring as body size increases. The reason for the difference in shape of the apex of the Md between adult females (pointed) and males (blunt) is not clear.

An obvious change was observed in the cross-section of the stylet fascicle during development and particularly between the nymphal and adult stages. Similar variation has been observed in other species^35^,^53^, although no data are yet available to determine whether the change is related to food or other environmental factors. The shape and position of the food and salivary canals are similar in nymphs and adults, while their size varies. It has been shown previously that the relative diameters of food and salivary canals are not directly related to the type of feeding^53^, nor is the size of the salivary canal correlated with the amount of saliva produced and the type of feeding^1^. Elson^54^ argues that mouthpart structure depends on the kind of food utilized, but Koteja^55^ found that internal mouthpart structures of scale insects representing different feeding types are similar. In addition, Tavella and Arzone compared three different species of Cicadellidae that are primarily phloem-, xylem- and mesophyll-feeding, respectively, and no obvious difference in internal structure was found^10^. Miyamoto^56^ suggested that differences are predictable based on phylogenetic relationships but much more comparative study will be needed to test this hypothesis.

The interlocking mechanism of the maxillary stylets prevents separation of the stylets during movement. The presence of three locks, which are formed by various processes, has been observed in Heteroptera^1^,^53^, Sternorrhyncha^57^, Fulgoromorpha^36^,^58^ and Coleorrhyncha^59^. Only two locks, composed of straight, hooked or T-shaped processes, have been observed in Cicadomorpha^35^, The number of locks provides an interesting insight into the phylogenetic relationships among these groups, suggesting a relatively distant relationship between Cicadomorpha to the other hemipteran suborders. The row of nodes and corresponding indentations on the middle ridge in adults may function as a rachet device for positioning the stylets in apposition to each other^11^.

The present study provides a basis for further comparative study of the mouthparts of cicadas and related groups of Hemiptera. Similar observations of different species are needed in order to determine the extent of variation in the observed structures among species and higher taxa.

## Materials and Methods

### Insect collecting and rearing

All Cicadidae used in this study were collected or reared at the campus of Northwest A&F University, Yangling, Shaanxi Province, China (34°160′ 56.24″N, 108°40′27.95″E).

First-instar nymphs. Adult female cicadas were collected in August 2014 and raised on the branches of *Pyrus xerophila* in an insect rearing cage. About one month later, eggs were removed from twigs and placed in a dish (50 mm in diameter, 10 mm in depth) in sealed plastic pots (150 mm in diameter, 60 mm in depth) and were moisturized by wet cotton. The eggs were kept at 8 °C under a photoperiod of 16:8 (L:D) h for 2 months. In November 20, 2014, the eggs were removed to room temperature to promote hatching. In May 20, 2015, the first-instar nymphs were obtained for morphological study^47^.

Second to fifth-instar nymphs were collected by digging beneath *Populus tomentosa* Carr. in May 2014, transferred into a beaker, cleaned with phosphate buffered saline and then preserved in 70% ethanol at 4 °C. Adults were collected by light trap in August 2014 and preserved in 70% ethanol at 4 °C.

### Scanning electron microscopy

The heads of cicada specimens were removed from the body with fine needles under a stereomicroscope (Olympus SZX10, Japan), and then rinsed twice for half a minute with 70% ethanol using an ultrasonic cleaner. Samples were then dehydrated in a graded series of 75%, 80%, 85%, 90%, 95% ethanol for 20 min each and 99.9% ethanol for 30 min twice before being transferred to a mixed solution of ethanol and tert-butanol (3:1, 1:1, and 1:3, by volume) for 15 min each, and finally to 100% tert-butanol for 30 min. Then the samples were placed into a freeze-drier (VFD-21S, SHINKKU VD, Japan) for 3 h. The dried sections of mouthparts were then mounted on the aluminum stubs with double-sided copper sticky tape and coated with gold/palladium (40/60) in a high resolution sputter coater (MSP-1S, Hitachi, Tokyo, Japan). The samples were subsequently examined with a Hitachi S-3400N SEM (Hitachi, Tokyo, Japan) operated at 15 kV. Ten individuals of each nymphal instar, and of male and female adults, were observed.

### Image processing and morphometric measurement

Photographs and SEMs were observed and measured after being imported into Adobe Photoshop CS6 (Adobe Systems, San Jose, CA, USA). The length of mouthparts was measured from the base of the first labial segment to the end of the third segment following Ruttner^60^. The width and height of each labial segment were measured from the middle part of each segment. Statistical analysis was conducted using one-way analysis of variance (ANOVA) followed by the Student-Newman-Keuls (SNK) test. Statistical analyses were executed using SPSS 19.0 (SPSS, Chicago, IL). Graphs were fitted by SigmaPlot 12.0 (Systat Software, San Jose, CA, USA). Schematic diagrams were drawn with Microsoft office Word 2007 and processed with Photoshop CS6. The sensilla are classified according to their external morphology, distribution and the presence or absence of pores. The terminology of sensilla follows Altner and Prillinger^43^ with more specialized nomenclature from Brożek and Bourgoin^44^.

## Additional Information

**How to cite this article**: Hao, Y. *et al*. Development of mouthparts in the cicada *Meimuna mongolica* (Distant): successive morphological patterning and sensilla differentiation from nymph to adult. *Sci. Rep.* **6**, 38151; doi: 10.1038/srep38151 (2016).

**Publisher's note:** Springer Nature remains neutral with regard to jurisdictional claims in published maps and institutional affiliations.

## Figures and Tables

**Table 1:** Distribution and morphometric data of various sensilla in different stage of *Meimuna mongolica*.

	N1				N2				N3				N4				N5				Adult			
	Distribution	Length (μm)	Basal Diameter (μm)	N	Distribution	Length (μm)	Basal Diameter (μm)	N	Distribution	Length (μm)	Basal Diameter (μm)	N	Distribution	Length (μm)	Basal Diameter (μm)	N	Distribution	Length (μm)	Basal Diameter (μm)	N	Distribution	Length (μm)	Basal Diameter (μm)	N
Str 1	Lb3	73.4 ± 2.3	3.5 ± 0.4	9	Lb3	89.7 ± 2.5	4.7 ± 0.2	7	Lb3	154.1 ± 18.5	6.8 ± 0.8	6	Lb3	254.8 ± 20.5	13.0 ± 0.8	5	Lb3	410.7 ± 39.5	17.4 ± 1.0	10	Lb3	247.1 ± 25.1	12.6 ± 0.2	10
Str 2	Lb	35.1 ± 0.9	2.7 ± 0.2	6	Lb	52.6 ± 5.9	3.3 ± 0.4	5	Lb	92.2 ± 17.8	6.0 ± 0.3	5	Lb	110.8 ± 24.3	6.5 ± 0.8	6	Lb	248.2 ± 38.4	10.1 ± 0.4	10	Lm, Lb	193.1 ± 14.2	5.2 ± 0.2	10
Str 3																					Lm, Lb	105.2 ± 7.8	4.7 ± 0.3	10
Str 4																					Lb	80.2 ± 2.5	4.4 ± 0.3	10
Sb 1	Lb	13.1 ± 1.4	1.9 ± 0.1	9	Lb	10.8 ± 2.3	2.6 ± 0.3	6	Lb	19.7 ± 3.0	3.6 ± 0.5	8	Lb	48.9 ± 19.8	7.0 ± 1.7	4	Lb	87.4 ± 13.7	10.3 ± 1.1	9	Lb	38.2 ± 3.3	4.5 ± 0.5	6
Sb 2	Lb3-D	8.0 ± 0.3	2.2 ± 0.1	6	Lb3-D	13.1 ± 1.0	2.7 ± 0.2	4	Lb3-D	21.3 ± 0.9	4.4 ± 0.3	4	Lb3-D	45.6 ± 2.8	7.6 ± 1.1	3	Lb3-D	58.7 ± 2.5	9.3 ± 0.5	7	Lb3-D	46.4 ± 1.9	8.1 ± 0.3	8
Sb 3	Lb2-V	27.3 ± 1.2	3.2 ± 0.2	6	Lb2-V	26.6 ± 1.5	3.8 ± 0.1	4	Lb2-V	44.5 ± 4.7	6.6 ± 0.2	4	Lb2-V	47.2 ± 4.0	7.9 ± 0.6	4	Lb2-V	78.0 ± 6.6	10.7 ± 0.3	3	Lb2-V	59.0 ± 6.1	7.6 ± 0.4	4
Sb 4	SF	19.8 ± 1.1	1.9 ± 0.1	9	SF	15.6 ± 2.8	2.3 ± 0.2	7	SF	20.1 ± 2.4	3.2 ± 0.3	7	SF	30.6 ± 4.2	3.9 ± 0.4	4	SF	48.1 ± 12.1	8.0 ± 1.0	8	SF	96.1 ± 10.2	8.3 ± 0.6	5
Sb 5																					Lb1,2-D	7.1 ± 1.2	2.1 ± 0.1	10
Sco1									Lb3		7.4 ± 1.6	3	Lb3		8.7 ± 0.4	9	Lb3		7.9 ± 0.5	5	Lb	1.0 ± 0.1	6.5 ± 0.2	10
Sco2																					Lb3	9.3 ± 0.4	5.7 ± 0.4	6
Hs																					Lb3		4.2 ± 0.3	10
Ps									Lb		1.7 ± 0.5	7	Lb		2.3 ± 0.3	5	Lb		2.0 ± 0.3	9	Lm, Lb		1.6 ± 0.1	10
Fls	SD	17.9 ± 0.7	3.1 ± 0.1	4	SD	16.4 ± 0.1	4.2 ± 0.1	4	SD	18.4 ± 2.2	5.4 ± 0.1	3	SD	23.1 ± 1.5	6.8 ± 0.2	3	SD	23.3 ± 2.4	7.3 ± 0.2	3				
Scs																					Lm, Lb3	9.2 ± 0.6	1.0 ± 0.2	10
Mt 1									Lb	3.6 ± 0.5	2.1 ± 0.3	8	Lb	4.3 ± 0.5	2.9 ± 0.4	6	Lb	4.0 ± 0.8	2.0 ± 0.3	4	Lb1	5.8 ± 0.4	2.4 ± 0.2	5
Mt 2																					Lb2-D	27.6 ± 3.7	21.4 ± 4.8	7
Mt 3																					Lb2-V	3.1 ± 0.6	1.4 ± 0.1	5
Mp																					Lb2,3	21.7 ± 2.4	33.3 ± 3.3	10

Data are means ± SE values obtained from scanning electron microscopy. N1–N5, first to fifth nymphal instar; N = sample number; Str1-4, sensilla trichodea I-IV; Sb1-5, sensilla basiconica I-V; Sco1-2, sensilla coeloconica I-II; Hs, hemispheric sensilla; Ps, poriform sensilla; Fls, finger-like sensilla; Scs, scaly structure; Mt1-3, microtrichia I-III; Mp, mammillary processes; Lm, labrum; Lb, labium; Lb1, 2, 3, the first, second, third segment of labium; Lb1, 2-D, the dorsal surface of the first and second labial segment; Lb2-V, ventral surface of the second labial segment; SD, dorsal sensory field on the labial tip; SF, sensory field on the labial tip.

**Table 2:** The morphometric data of mouthparts of *Meimuna mongolica*.

		N1 (μm)	N2 (μm)	N3 (μm)	N4 (μm)	N5 (μm)	Adult (μm)	F	*P*
Lm	Length	46.3 ± 1.2 c	62.3 ± 2.3 c	104.2 ± 0.3 c	163.6 ± 6.9 c	392.2 ± 30.3 b	1318.6 ± 104.2 a	129.0	0.0
	Width	35.2 ± 0.5 e	38.3 ± 0.3 e	79.1 ± 0.9 d	147.3 ± 5.6 c	238.1 ± 21.9 a	182.5 ± 6.4 b	95.1	0.0
Lb1	Length	85.0 ± 3.1 d	147.4 ± 17.8 d	283.7 ± 22.6 d	521.9 ± 59.0 c	900.3 ± 50.3 b	1444.4 ± 74.5 a	69.9	0.0
	Width	96.3 ± 4.8 d	148.9 ± 1.6 d	261.9 ± 21.3 c	404.5 ± 74.1 b	772.9 ± 35.6 a	425.6 ± 15.6 b	65.9	0.0
Lb2	Length	88.3 ± 13.3 d	153.9 ± 10.2 d	317.1 ± 55.0 d	541.4 ± 58.1 c	922.9 ± 58.3 b	1289.4 ± 41.8 a	81.1	0.0
	Width	91.8 ± 7.4 e	152.9 ± 3.7 d	238.9 ± 27.1 c	320.9 ± 5.2 b	603.7 ± 15.9 a	632.6 ± 12.4 a	235.2	0.0
Lb3	Length	347.5 ± 20.9 e	598.4 ± 20.9 e	1189.2 ± 35.9 d	2320.8 ± 78.3 c	3748.8 ± 55.4 b	6005.9 ± 86.7 a	774.4	0.0
	Width	68.6 ± 4.6 e	101.6 ± 0.5 e	175.9 ± 12.0 d	265.3 ± 2.9 c	419.9 ± 18.3 b	470.9 ± 9.9 a	151.9	0.0

Data are means ± SE values obtained from scanning electron microscopy. Means in the same row followed by different letters (a–e) are significantly different (SNK test, *P* < 0.05). N1–N5, the first to fifth instar nymphs; Lm, labrum; Lb1, the first segment of labium; Lb2, the second segment of labium; Lb3, the third segment of labium.
